# Supplementary material for: Cerebral organoids in primary progressive multiple sclerosis reveal stem cell and oligodendrocyte differentiation defect
Source: Biol Open. 2023 Mar 6;12(3):bio059845. doi: 10.1242/bio.059845 (PMC10040243; doi:10.1242/bio.059845)
Supplement: Supplementary information [file biolopen-12-059845-s1.pdf]

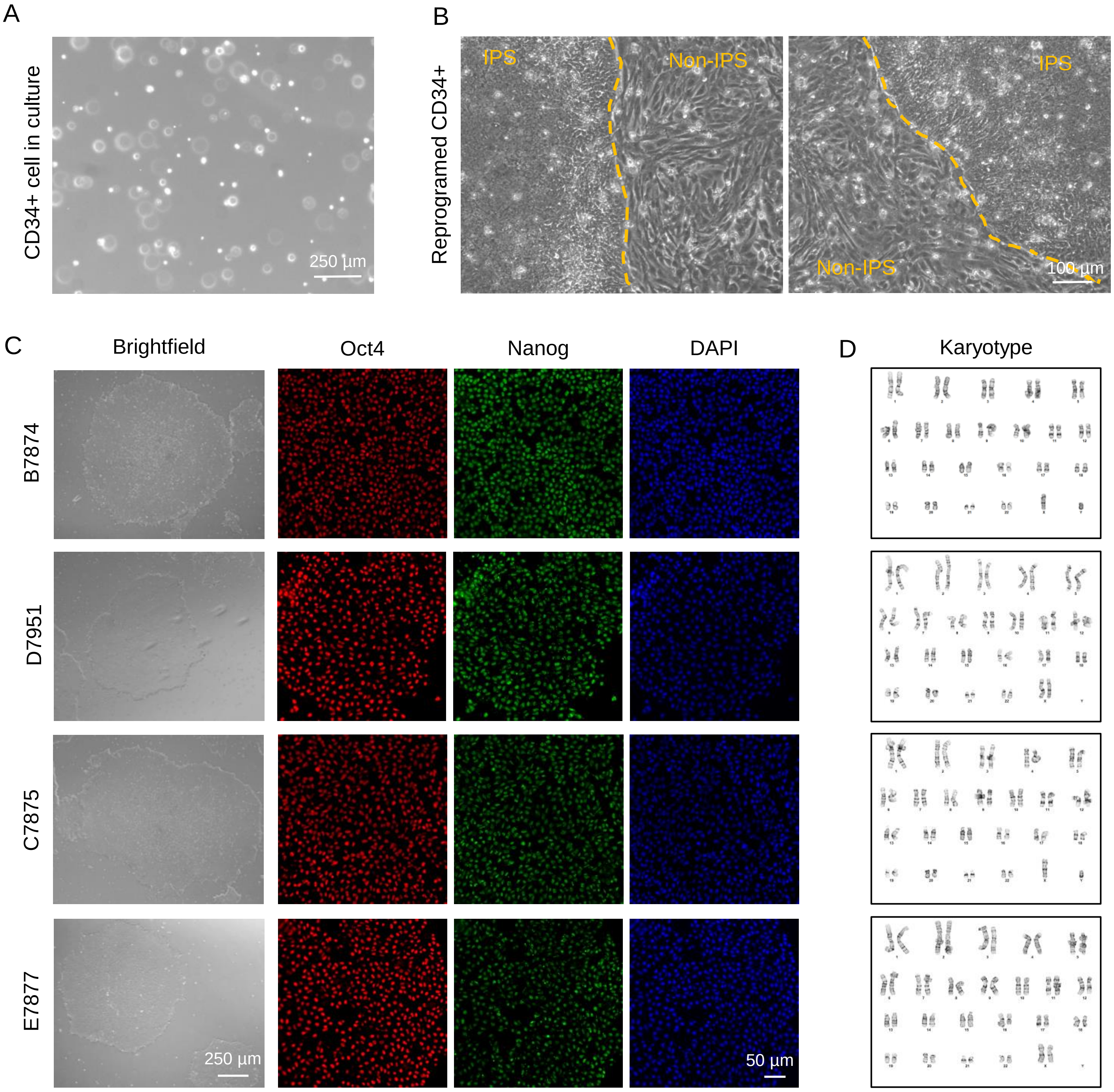

Fig. S1. CD34+ derived IPS cells characterization and quality control.

A) Brightfield picture of patient CD34+ cells in expansion.

B) Representative images of IPS cell colonies growing 20 days after reprogramming. iPSCscolonies were manually scraped and replated. The yellow dotted line shows the distinction between iPSCs and cells that failed to reprogram.

C) Characterization of iPSCs pluripotency markers. Left panel shows brightfield picture of IPScells. Right panels show immunofluorescence for pluripotency markers Oct4, Nanog and the DAPI counterstain.

D) Karyotype analysis of CD34+ derived iPSCs, all display normal karyotype.

Biology Open • Supplementary information

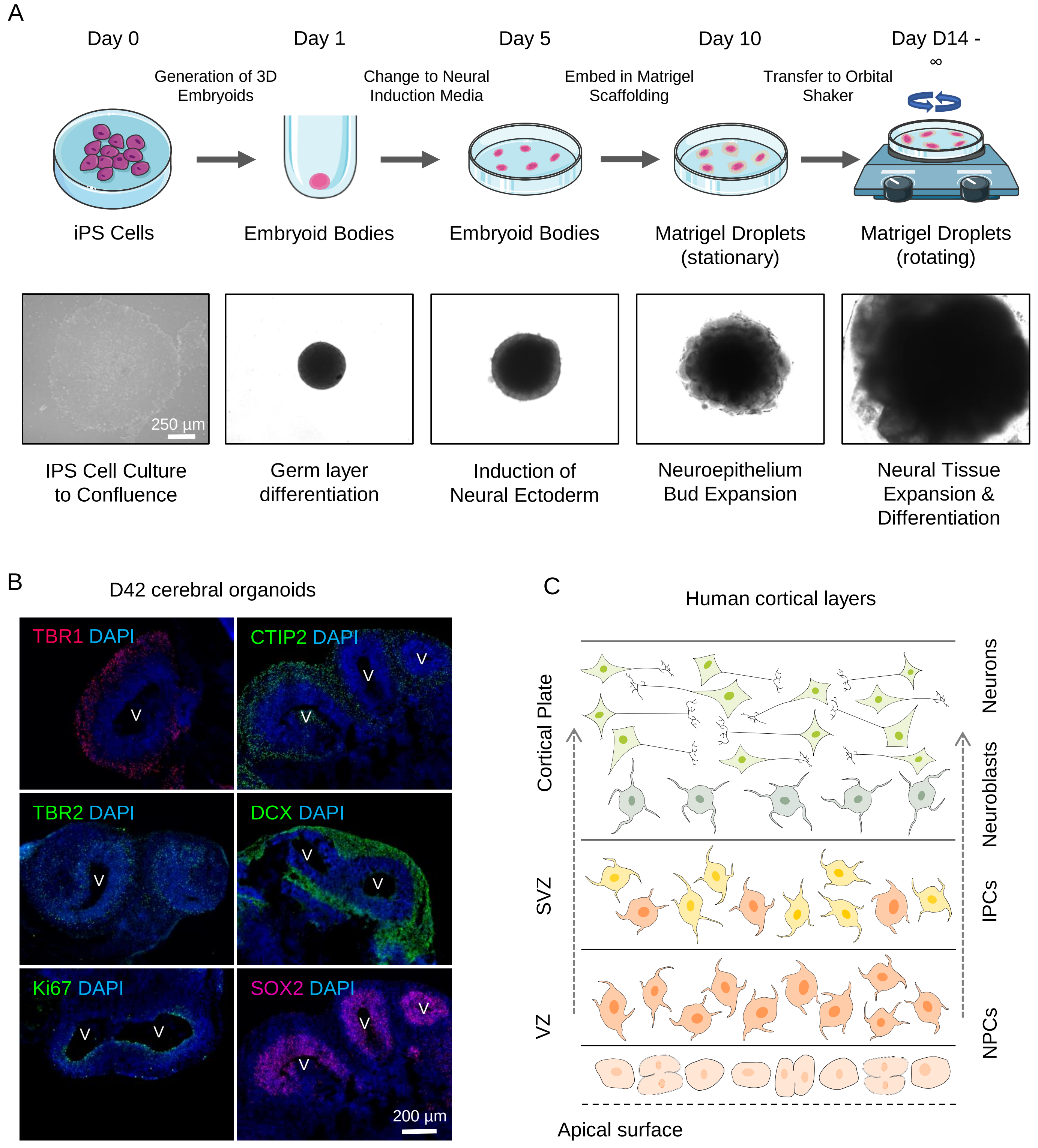

Fig. S2. C-organoids protocol and cell population characterization.

A)Schematic representation (top panels) and brightfield pictures (bottom panels) of the mainsteps of the c-organoid derivation protocol.

B)Immunofluorescence images of c-organoids at D42 demonstrate ventricle-like structure (V)aligned with neural precursors and their progeny at different stages of differentiation. Ki67 marker represents proliferating cells, SOX2 for neural stem cells, TBR2 for intermediate progenitors, DCX for neuroblasts, TBR1 for early-born cortical neurons and CTIP2 for deep-layer subcortical neurons. DAPI for nuclear counterstaining.

C)Schematic representation of the cortical structure found in human c-organoids. The ventricularzone containing the stem cell pool and most proliferating cells, the SVZ containing mostly IPCs and the oSVZ and CP containing neuroblasts and mature neurons.

Biology Open • Supplementary information

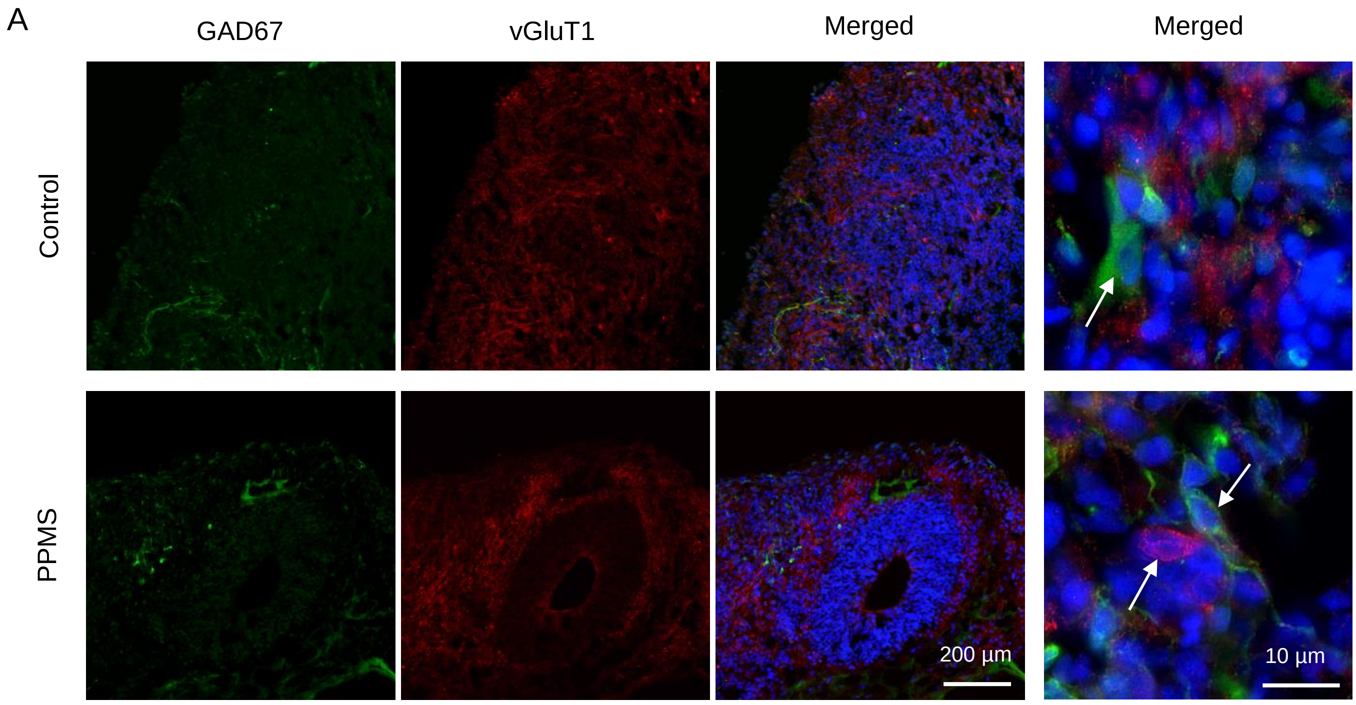

Fig. S3. Glutamatergic and GABAergic neurons expression.

A) Pictures of immunofluorescence against GABAergic (GAD67) and Glutamatergic (vGluT1) neuronal markers taken by confocal microscopy in control (top) and PPMS (bottom) in c-organoids at D42. The right panels show higher magnifications. White arrows show positive cells.

Biology Open • Supplementary information
